# Computational Investigation of Vessel Injury Due to Catheter Tracking During Transcatheter Aortic Valve Replacement

**DOI:** 10.1007/s10439-024-03462-8

**Published:** 2024-04-08

**Authors:** David G. Symes, Laoise M. McNamara, Claire Conway

**Affiliations:** 1 Biomedical Engineering, School of Engineering, College of Science and Engineering, University of Galway, Galway, Ireland; 2 Department of Anatomy and Regenerative Medicine, Royal College of Surgeons in Ireland (RCSI), Dublin, Ireland; 3 Trinity Centre for Bioengineering, Trinity College Dublin & RCSI, Dublin, Ireland

**Keywords:** Finite element analysis, Catheter trackability, Atherosclerosis, Aorta, Transcatheter aortic valve replacement, Plaque rupture

## Abstract

Catheter reaction forces during transcatheter valve replacement (TAVR) may result in injury to the vessel or plaque rupture, triggering distal embolization or thrombosis. In vitro test methods represent the arterial wall using synthetic proxies to determine catheter reaction forces during tracking, but whether they can account for reaction forces within the compliant aortic wall tissue in vivo is unknown. Moreover, the role of plaque inclusions is not well understood. Computational approaches have predicted the impact of TAVR positioning, migration, and leaflet distortion, but have not yet been applied to investigate aortic wall reaction forces and stresses during catheter tracking. In this study, we investigate the role that catheter design and aorta and plaque mechanical properties have on the risk of plaque rupture during TAVR catheter delivery. We report that, for trackability testing, a rigid test model provides a reasonable estimation of the peak reaction forces experienced during catheter tracking within compliant vessels. We investigated the risk of rupture of both the aortic tissue and calcified plaques. We report that there was no risk of diseased aortic tissue rupture based on an accepted aortic tissue stress threshold (4.2 MPa). However, we report that both the aortic and plaque tissue exceed a rupture stress threshold (300 kPa) with and without the presence of stiff and soft plaque inclusions. We also highlight the potential risks associated with shorter catheter tips during catheter tracking and demonstrate that increasing the contact surface will reduce peak contact pressures experienced in the tissue.

## Introduction

Aortic stenosis (AS) is defined as a degenerative disease that develops from the build-up of calcium deposits on the aortic valve. There is an increasing risk of cardiovascular diseases such as severe AS because of the ever-increasing elderly population in developed countries, with an estimated incidence of 3–4% in those aged over 75 [1–3]. Severe asymptomatic AS has a mortality rate of between 33 and 50% in the first 2 years in untreated patients [4] and has a per annum cost of ~ $10 billion to the US economy in terms of total patient care [5]. It has commonly been treated via open-heart surgical aortic valve replacement (SAVR) [6] for younger patients (<75 years) [7]. However, 40% of patients are deemed unsuitable for SAVR due to operating risks [3], such as older patients (>75 years) [7], patient frailty, restricted mobility, and porcelain aorta [8]. These patients are generally recommended for treatment via the minimally invasive transcatheter aortic valve replacement (TAVR) procedure. During TAVR, the valve device is delivered transfemorally via a catheter, which is navigated through the aortic arch to the aortic valve for treatment. Approximately half of the strokes that occur after TAVR are periprocedural (within 48 h of TAVR) and are embolic in nature [9–11].

Catheter tracking through the aortic anatomy during TAVR delivery is a potential cause of vessel and plaque injury. Clinically, catheter tracking and positioning are assessed via radiographic markers on the catheter through fluoroscopy, which cannot provide information regarding the forces and stresses imparted on the vessel wall and plaques. As such, the forces that are being imparted during tracking are assessed by “feel” from physicians, as well as through in vitro testing. In vitro silicone benchtop anatomies are commonly used to assess catheter performance. However, in vitro benchtop models can be limited in terms of their ability to predict in vivo reaction forces, primarily because a commonly used material for these models (Delrin) has a stiffness of 2,300 MPa. The mechanical behaviour of this silicone typically does not capture the non-linear behaviour of an aortic wall or atherosclerotic plaques. In addition, in vitro testing of catheter delivery systems is not a standardized process, as each manufacturer generally defines their own trial and sampling methodology to evaluate catheter mechanical characteristics [12]. As such, variances between in vitro models and the intended use conditions can be a limitation of these models.

An in vivo study conducted by Capron and Bruneval investigated balloon catheter frictional forces in rat aortas over 30 days and found an increase in endothelial denudation, which in turn could lead to thrombosis [13]. Similarly, Caldwell et al. carried out an in vitro experiment with reciprocating frictional apparatus to examine frictional forces between phosphynylated and untreated catheters within porcine aorta [14]. The results found the removal of endothelial cells by both catheter types, however, the treated catheter had a lower coefficient of friction and allowed greater retention of endothelial cells [14]. The results highlight the risk of injury to the endothelial layers of the aorta or potential damage to existing atherosclerotic plaques during catheter tracking that may trigger thrombosis or distal embolization associated with ischaemia and infarction. The design of the catheter tip could also be a factor in potential plaque rupture. A study by Perna et al. investigated tip contact forces resulting in cardiac perforation in swine atria and found that forces between 1 and 2.5 N resulted in cardiac perforation [15]. However, this has not been investigated or quantified for the aorta wall or atherosclerotic plaques during TAVR delivery. Despite increasing clinical research on the effect that different replacement valves have on embolic debris generation [16], and studies considering the advantages, disadvantages, and development of embolic capture devices [17, 18], the specific mechanical environment that leads to thrombus and embolus formation during catheter tracking is not yet fully understood.

Within the ageing and diseased aorta, atherosclerotic plaques play a significant role in increasing overall arterial stiffness. In addition, cholesterol esters found in the necrotic core become crystalline over time, resulting in microcalcifications forming, which in turn leads to further stiffening of the atherosclerotic plaque. However, defining the mechanical stiffness of plaques is difficult due to the wide variability in mechanical stiffness between plaques containing discrete calcified volumes of varying size and shape (0.1–10 MPa) [19] and larger “bulk” calcifications (10 Gpa) [19]. This is primarily due to the number of microcalcifications present. Defining the stress at which plaque rupture occurs has been equally challenging with a previous study by Lendon et al. reporting a rupture stress threshold of 300 kPa [20]. Studies by Moldonado et al. and Kelly-Arnold et al. propose that plaque rupture may initiate due to plaque cavitation, which occurs when voids in the plaque undergo explosive growth due to large tensile loads [21–23]. This is hypothesized to occur when aortic tissue stresses exceed $0.83\dot{3}$ *E*_*t*_, where *E*_*t*_ is the Young’s Modulus of the tissue [21–23]. While this approach does not consider the non-linear mechanical response of plaques, it presents a useful comparative metric to assess the risk of plaque rupture leading to either thrombus or embolus formation.

Finite element (FE) analysis is a valuable predictive tool that has been applied to predict outcomes during and after TAVR. Sun et al. and Gunning et al. implemented FE methods to investigate leaflet deformation during deployment [24, 25]. Tzamtzis et al. conducted a study to assess and compare the radial forces produced by self-expanding and balloon-expanding valvular devices [26]. They found that the native geometry and stiffness were determining factors on the predicted outcome of radial forces on the left ventricular outflow tract [26]. Two studies conducted by Capelli et al. and Morganti et al. analysed TAVR deployment within patient-specific anatomies to assess implantation feasibility, valve positioning, and the stress distribution caused by deployment geometry [27, 28]. FE modelling approaches have the potential to quantify aortic tissue and catheter stresses that are not possible to determine through in vitro analysis of TAVR performance. However, computational approaches have not been applied to comprehensively investigate how the delivery catheter imparts contact pressures and stresses on the aorta wall, or what consequence these pressures and stresses could result in, especially concerning thrombus and embolus formation.

As such, the aim of this study was to develop and analyse the tracking forces and stresses produced by an in-silico TAVR catheter model tracking around an idealized aortic arch anatomy to assess the potential risk catheters pose during TAVR intervention. An in vitro Delrin testing model is utilized for initial comparison with the in-silico model. The predicted tracking forces are compared with reaction force-displacement data from the in vitro Delrin testing model. A parametric study is then performed to understand the influence of varying aorta wall stiffnesses and biomechanically representative boundary conditions on reaction forces. Different catheter tip lengths are examined to determine their impact on aortic wall contact pressure during catheter tracking. Lastly, idealized plaque regions are introduced to the aorta wall. The aortic wall stresses-induced during catheter tracking are then assessed to determine the potential risk of vessel injury or plaque rupture leading to thrombus or embolism formation.

## Materials & Methods

### Catheter Delivery System and Idealised Aortic Wall

**Fig. 1:**
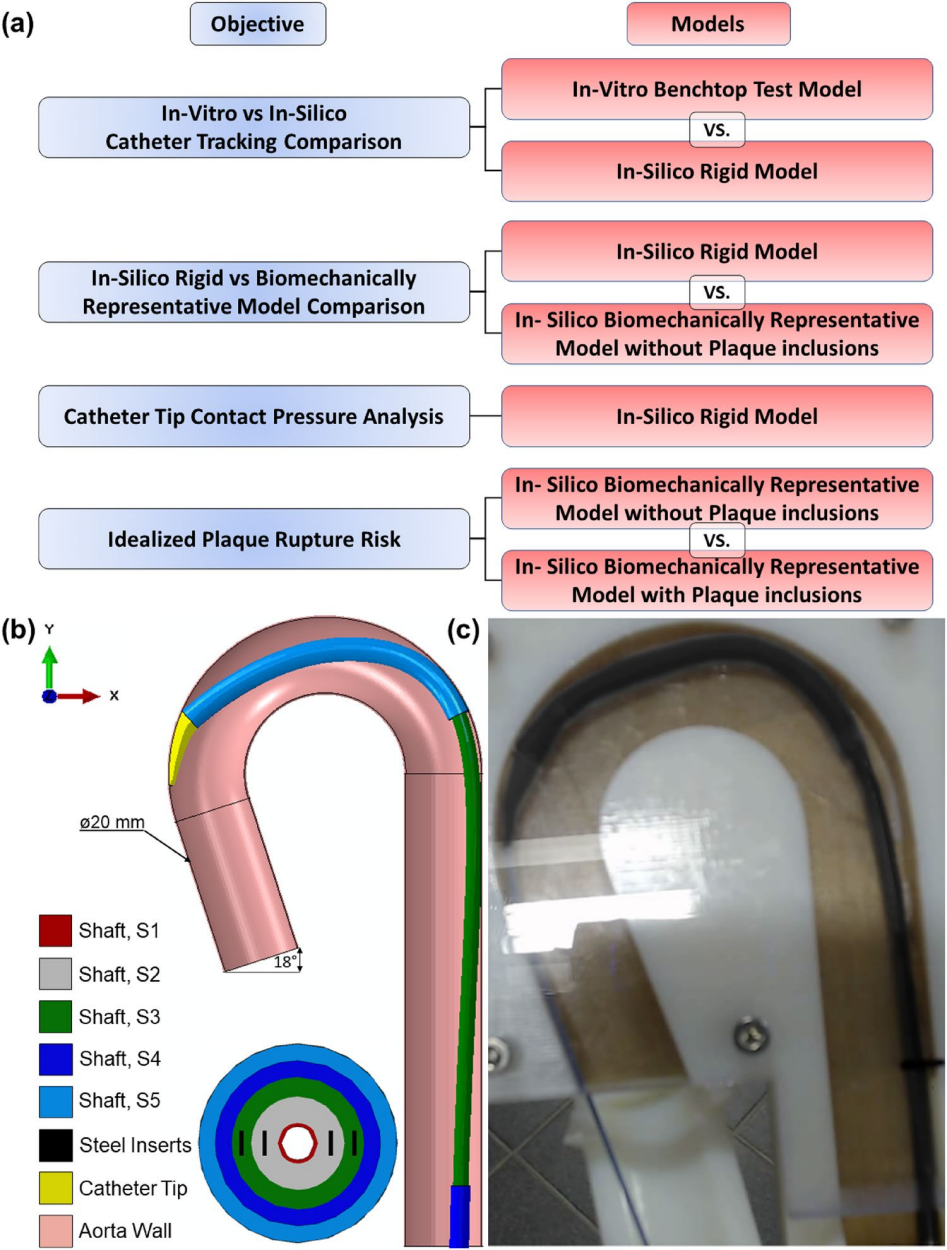
**a** Flowchart linking study objective with aorta model type analysed, **b** 3D representation and measurements of idealized aortic arch and catheter material shafts identified per Table 1; **c** Anonymised (outline of morphology—edited per request) benchtop in vitro model during catheter trackability testing

A commercially available delivery catheter system is studied, which is comprised multiple separate shafts [organised radially, S1–S5 per the sketch below (Fig. 1)] and the catheter tip. The dimensions of each catheter shaft are taken from the nominal specification, and the mechanical properties are derived from experimental 3-point bend-testing of the physical system using a Zwick uniaxial tester. Each shaft, in isolation, had mechanical properties implemented and reproduced using an in-silico 3-point bend test simulation. Table 1 provides the mechanical properties of each shaft (S1–S5) and the catheter tip. The shaft, S3 also behaves orthotropically, which is captured in Table 1. There are steel wires (Young’s Modulus, *E* = 180 GPa) embedded within the walls of the S2 and S3 shafts and nitinol wires (*E* = 70 GPa) within the S5 shaft. The replacement aortic valve is placed within shaft S5 before delivery. The presence of the valve results in a doubling of the Young’s Modulus in this region. The delivery system was meshed using the Abaqus/Explicit FE solver (SIMULIA, v2020) with 3D reduced integration linear continuum hexahedral elements, with a mesh density of ~ 70,000 elements, and assumed to be primarily an isotropic and linear elastic material.

**Table 1:** Nominal specifications for each catheter shaft dimension (presented in Fig. 1*)* and the material parameters characterising the elastic response of each delivery system shaft derived from experimental 3-point bend-testing using a Zwick uniaxial tester*.*

Shaft	ID (mm)	OD (mm)	Length (mm)	Shaft E (MPa)	Shaft G (MPa)		Poisson’s ratio, v
S1	1.0	1.22	260	~ 7,000	N/A	0.3	
S2	1.22	1.82	180	~ 800	N/A	0.3	
S3	1.82	4.0	180	~ 350	~ 4,000	0.3	
S4	4.0	5.0	60	~ 300	N/A	0.3	
S5	1.22	6.0	80	~ 100–200	N/A	0.3	
Tip	1.0	1.0–6.0 (taper)	18	6	N/A	0.3	

The idealized aortic arch modelled in this study was established from a custom benchtop aorta anatomy model which has been used in industry for in vitro catheter testing assessing trackability performance. The dimensions and curvature of the in-silico arch are representative of the bespoke experimental Delrin (Acetal) aorta model and are presented in Fig. 1b. The inner vessel has a diameter (ID) of 20 mm, representing the narrowest aorta arch diameter sizes reported in ageing male and female patients [29, 30]. This study sought to predict the reaction forces experienced during in vitro trackability testing, whereby the catheter is advanced through the chosen pathway (in this instance, the descending aorta, through the arch and into the ascending aorta). The rigid in-silico arch is then converted to a 3D solid deformable aorta to analyse reaction forces and stresses within a biomechanically representative arch. The addition of a non-stick coating (e.g., Teflon) is one experimental approach used to reproduce as closely as possible the in vivo frictional behaviour of the catheter tracking through the aorta and around the arch. An in-silico arch model was first defined as a discrete rigid shell model, for comparative purposes with the experimental Delrin in vitro model (Fig. 1c). This idealized shell aortic model’s wall was meshed with 3D bilinear rigid quadrilateral elements, with a mesh density of ~ 44,000 elements.

Regarding mechanical properties, the biomechanically representative aortic arch model was assigned varying stiffnesses (5 and 8 MPa), representing ageing and diseased aortic tissue [31–33], for comparison with the rigid in-silico simulations. A thin outer layer of shell elements (4-node reduced integration) was also included to surround the idealized arterial models to represent the physiological environment in which the aorta would be embedded. A similar approach has been applied in previous studies by Conway et al. and Harewood et al. so that physiologically representative boundary conditions could be applied to the artery [34, 35]. This thin outer layer surrounding the arterial vessel was considered to be isotropic and linear elastic with a stiffness of *E* = 1 MPa, a Poisson’s Ratio of *ν* = 0.45, and an assigned thickness of 0.1 mm.

### Catheter Tracking Investigation

The Abaqus/Explicit FE solver (v2020, DS Simulia, USA) was used to model the catheter delivery system tracking around the idealized aortic arch geometry. The idealized rigid model was first constrained in all three translational directions to simulate the fixed benchtop in vitro model. The idealized biomechanically representative arch model was developed by constraining the ascending and descending ends of the aorta to anchor the aorta geometry during deformation. The remaining aorta wall was free to deform in all directions. The proximal and distal ends of the layer of shell elements surrounding the arterial vessel were similarly constrained, which reduced large unrealistic deformations in the aorta while still allowing the aorta to move and deform when contacted by the catheter during tracking. The delivery system was fixed at its base in the x and z directions, a translational displacement of 140 mm was applied to the base of the delivery system in the y-direction to simulate tracking. The remaining catheter body was free to translate in all degrees of freedom.

Contact with the aorta wall was simulated by defining general contact with surface pairs between the catheter (slave) and the aortic wall (master). “Hard” contact was defined in this contact model to minimize the penetration of the slave surface (catheter) into the master surface (aortic wall) and prevent tensile stress across the interface. The in vitro anatomy and catheter were coated to mimic in vivo frictional behaviour conditions resulting in a coefficient of friction of 0.05 [36], which was then implemented in all catheter tracking simulations in this study. The ratio of kinetic energy to internal energy was monitored and remained less than 5% for the majority of each simulation.

### Investigation of the Role of Catheter Tip Length

**Fig. 2:**
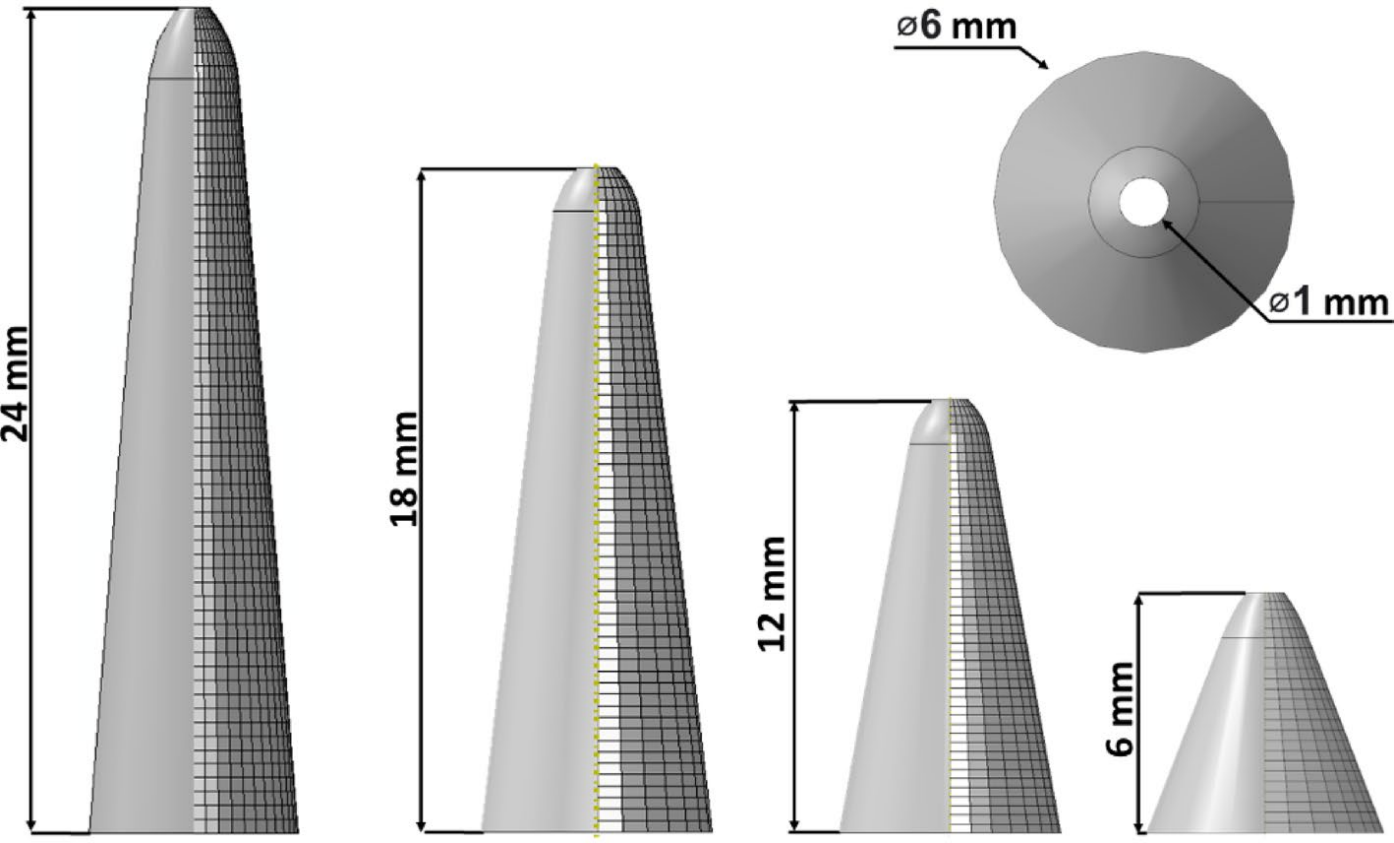
2D representation and measurements of varying idealized tip geometries that are used to investigate the impact catheter tip lengths have on reaction forces and contact pressures during tracking

The influence of catheter design, namely tip length, on reaction forces, contact pressures and how that is linked to tip deflection angle during tracking was investigated in this study. Four catheter tip lengths were examined: 6 mm, 12 mm, 18 mm, and 24 mm, which are detailed in Fig 2. The 18 mm tip length is the same length used for comparison with the in vitro benchtop model. All tracking simulations for this investigation were carried out within the idealized rigid aorta model.

### Atherosclerotic Plaque Tissue

The model was further developed to include idealized embedded plaques. The atherosclerotic plaques were assumed to be similar to either the partially calcified fibrous tissue (soft plaques) or bulk calcifications (stiff plaques) reported in the study by Ebenstein et al. [19]. These idealized plaques were modelled as isotropic, homogeneous, and linear elastic. An elastic modulus of either *E* = 10 MPa or 10 GPa with a Poisson’s Ratio of *v* = 0.3 is applied to each soft and stiff plaque set, respectively [19]. The plaques were modelled as embedded element sets between two layers of elements to mimic sub-endothelial formation in the arch wall and were 1 element thick ($0.83\dot{3}$ mm). Idealized plaques are positioned in regions where there is the highest risk of damage due to the higher reaction forces experienced by the catheter in these regions. The predictions obtained from the idealized, embedded plaque model were analysed to determine the plaque rupture risk due to the stresses the delivery catheter system imparts on the arch. This study investigates two rupture stress threshold hypotheses for aortic tissue with and without plaque inclusions; (H1) suggests that explosive growth of small voids within the tissue could initiate rupture and occurs when aortic wall tissue stresses exceed $0.83\dot{3}$ *E*_*t*_ (where *E*_*t*_ is the aortic tissue’s Young’s Modulus; *E*_*t*_ = 5 MPa) [23]. Another study, by Lendon et al., proposed a plaque rupture stress threshold of 300 kPa [20] (H2).

### Catheter Tracking Analysis Metrics

**Fig. 3:**
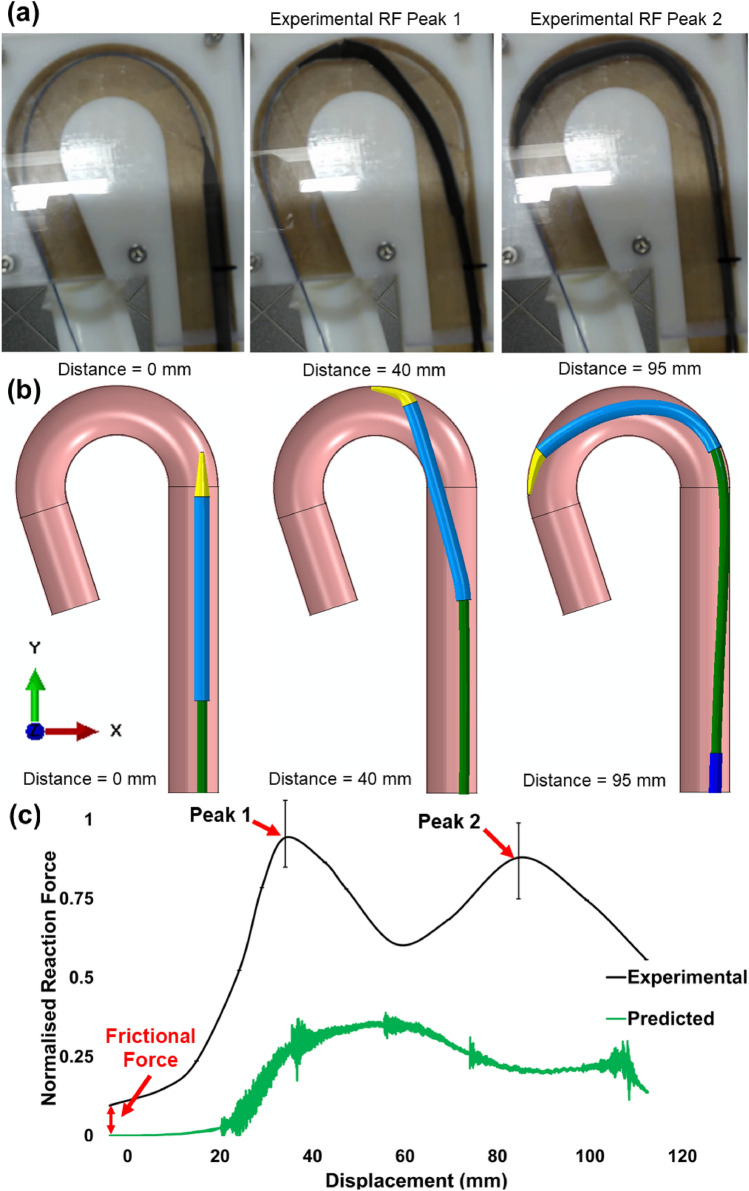
Stages of the catheter’s progress while tracking around the **a** in vitro test anatomy and **b** in-silico idealized aorta arch. Timepoints signify start point and in vitro test reaction force peaks presented in **c** Predicted temporal reaction force obtained from the FE simulation compared with in vitro reaction force data. All data are normalised against the first in vitro reaction force peak

In laboratory trackability experiments, the key output is the reaction force experienced at the proximal base of the catheter delivery systems [37]. As such, experimental reaction force data (*n* = 5, avg) obtained from the in vitro trackability test (Fig. 3a) were compared with the predicted reaction force data taken from the base of the in-silico catheter model (Fig. 3b) during tracking. Axial reaction force data were extracted from the nodes on the catheter’s base in the Abaqus/Explicit FEA solver before analysis. A custom Python postprocessing script was developed (similar to the analysis performed by Conway et al. [38]), to enable the plotting of high-resolution maximum principal stress histograms to investigate tissue states at specific tracking distances to the H1 or H2 hypotheses. All histograms are plotted as log element volume versus log maximum principal stress to capture the ranges of values.

## Results

### Reaction Force Predictions for Idealised Aortic Wall Models

**Fig. 4:**
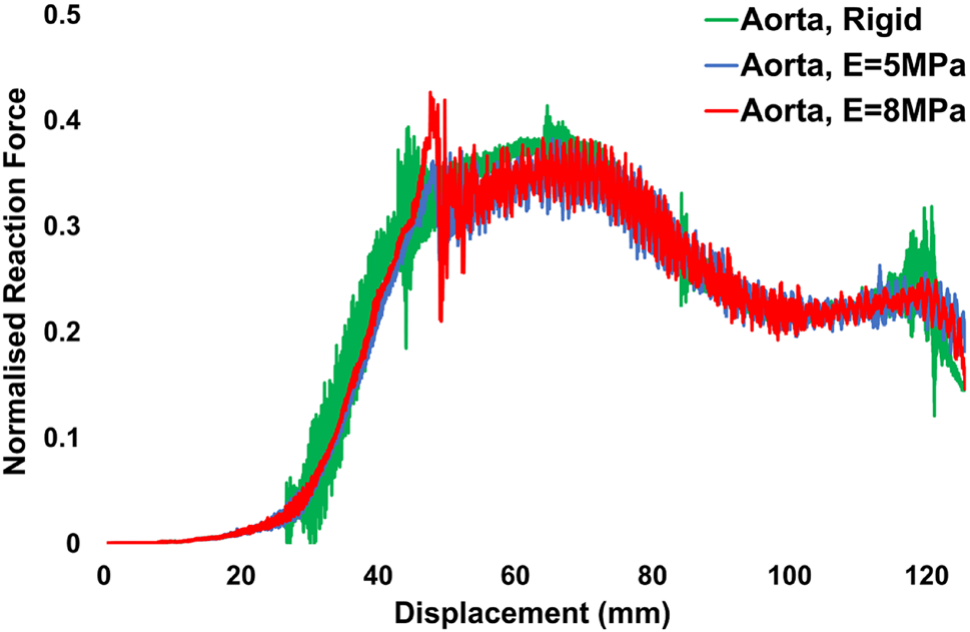
Predicted temporal reaction forces obtained from the rigid and biomechanically representative model simulations. All data are normalised against the first in vitro reaction force peak (see Fig. 3c)

Experimental reaction force data obtained from the in vitro trackability test (Fig. 3a) were compared with the predicted reaction force data taken from the base of the in-silico catheter model (Fig. 3b) during tracking. Fig. 3c presents an under-prediction of peak reaction forces between the computational simulation and the in vitro reaction force peaks. The in vitro force peaks occur at normalised values of 1 and 0.93 for peaks 1 (40 mm) and 2 (95 mm), respectively. The normalised predicted in-silico force peaks at 0.37 and occurs after 65 mm of tracking around the arch. All reaction force data are normalised to the first experimental force peak measured. An investigation was then performed to determine the variance in reaction force predictions between the rigid and the biomechanically representative aortic wall model (Fig. 4). The normalised peak reaction forces for “ageing and diseased” aortic wall models (5, 8 MPa) were similar to those predicted in the rigid model, 0.35 vs 0.37 at 65 mm of tracking, respectively.

### Predicted Plaque Rupture Risk

**Fig. 5:**
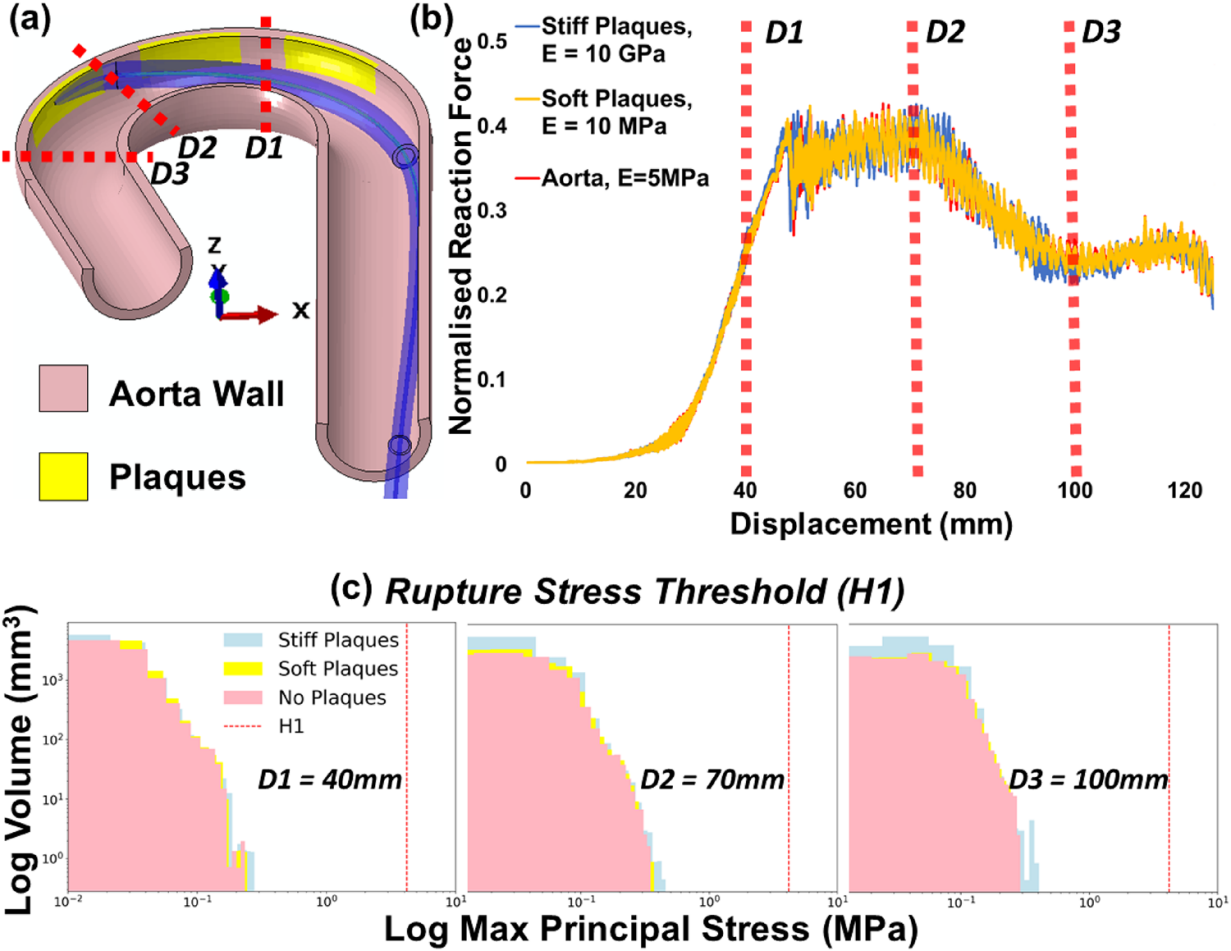
**a** Cross-section image of idealized aorta arch (pink) with plaques (yellow). Maximum principal stresses were analysed in the histograms at three key displacement time points, based on reaction force-displacement curves: D1 (distal upper arch), D2 (proximal upper arch), and D3 (proximal aorta arch). **b** Predicted temporal reaction forces obtained from simulations with the biomechanically representative model with no plaques (5 MPa) and the model with stiff and soft plaque inclusions. All data are normalised against the first in vitro reaction force peak (see Fig. 3c). **c** Histograms of logarithmic max principal stress per logarithmic element volume of the aorta arch wall without plaque inclusions at the displacement points highlighted. The rupture stress threshold, *H1* = $0.83\dot{3}$ *E*_*t*_ = 4.1667 MPa

**Fig. 6:**
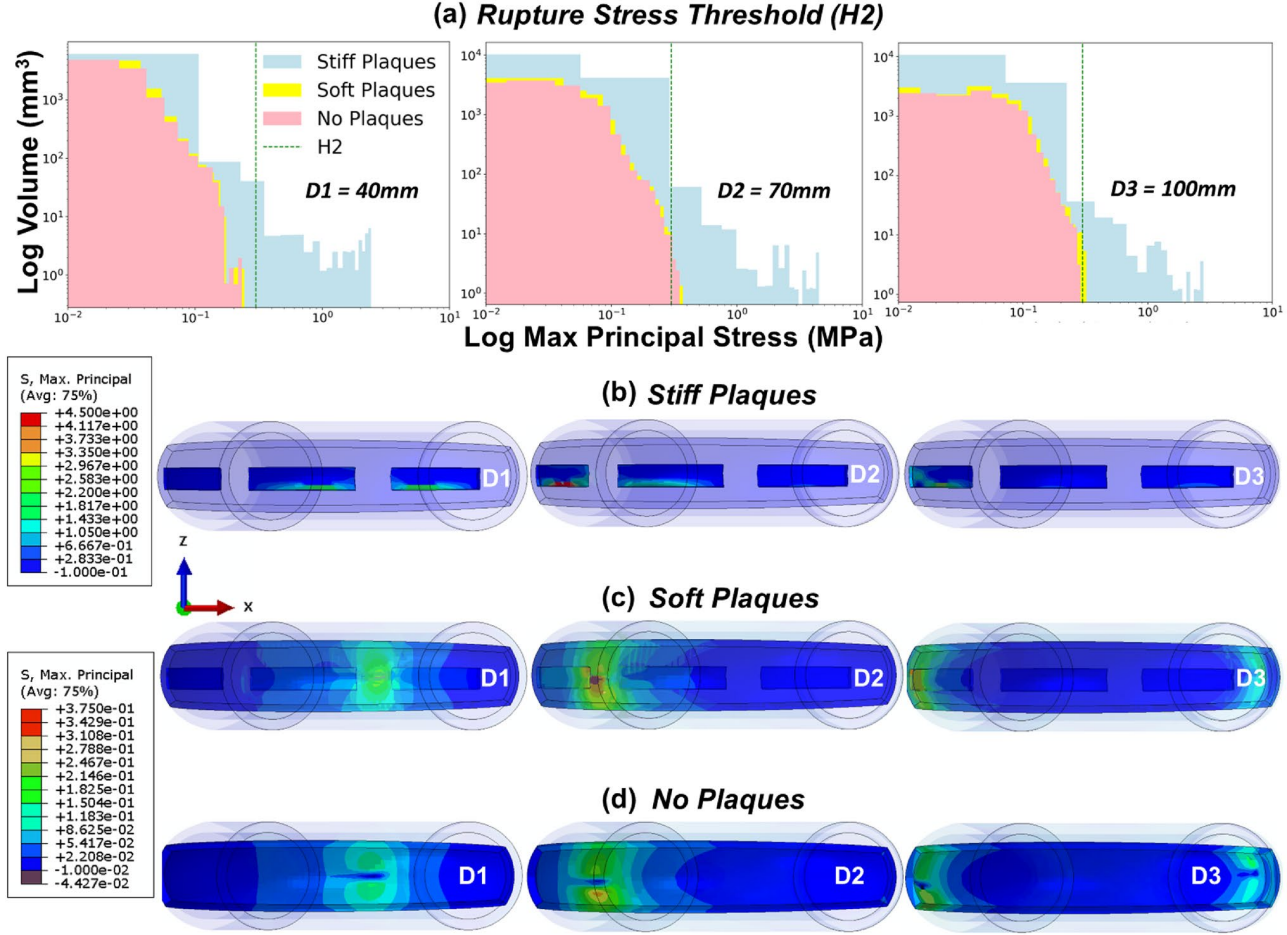
**a** Histograms of logarithmic maximum principal stress per logarithmic element volume of the aorta arch wall with plaque inclusions at the displacement points depicted in Fig. 5a. The rupture stress threshold, *H2* = 0.3 MPa; **b**–**d** Contour plots of max principal stress at key displacement points, see Fig. 5a*,* for the idealized aorta model with stiff plaques, soft plaques, and no plaques, respectively. The first legend corresponds to the stiff plaques only, the second legend corresponds to both the soft plaques and no plaque models

The max principal stresses in the aorta wall and the soft/stiff plaques while the catheter delivery system tracked through the aorta were analysed and compared to the plaque rupture risk thresholds (H1 & H2). Firstly, a threshold was defined (H1) based on a study by Kelly-Arnold et al. [23], where they hypothesise that aortic tissue rupture may occur at $0.83\dot{3}$ *E*_*t*_, where *E*_*t*_ is the Young’s Modulus of the aorta wall tissue (*E*_*t*_ = 5 MPa). As such, the rupture stress threshold for the aortic wall tissue surrounding the plaques was 4.1667 MPa. The second threshold, H2, employed a rupture stress threshold (0.3 MPa) for aortic tissue with plaque inclusions. Three models (stiff plaque, soft plaque or no plaques) were analysed in this study at three key displacements [D1; 40 mm, D2; 70 mm; D3; 100 mm (Fig. 5a, b)] for both H1 (Fig. 5c) and H2 (Fig. 6a). The aortic model with no plaques is the same aortic model that is presented in Fig. 4 (5 MPa wall stiffness).

Using the custom Python postprocessing script described previously, the extraction of deformed element volume and maximum principal stresses per element was performed. This enabled the plotting of high-resolution maximum principal stress histograms to investigate tissue states at specific tracking distances with respect to the H1 or H2 thresholds. Implementing this Python script, the stresses predicted to occur on the aortic wall tissue without plaque inclusions (aorta arch only, ascending and descending aorta excluded from analysis), with regards to H1 (Fig. 5) suggest no maximum principal stresses exceeded $0.83\dot{3}$ *E*_*t*_ at any key displacement points (D1–D3) for any model. However, when we analyse the same models with plaque inclusions for H2 (Fig. 6), we can see a risk of rupture for 0.65 to 1.1% of the element volume in the stiff plaque model at all three displacement points. The soft plaque model exceeds the threshold at D2 (0.075%) and D3 (0.05%), while the no plaque model exceeds this threshold at displacement point D2 (0.065%) only.

### In-Silico Predictions for Catheter Tip

**Fig. 7:**
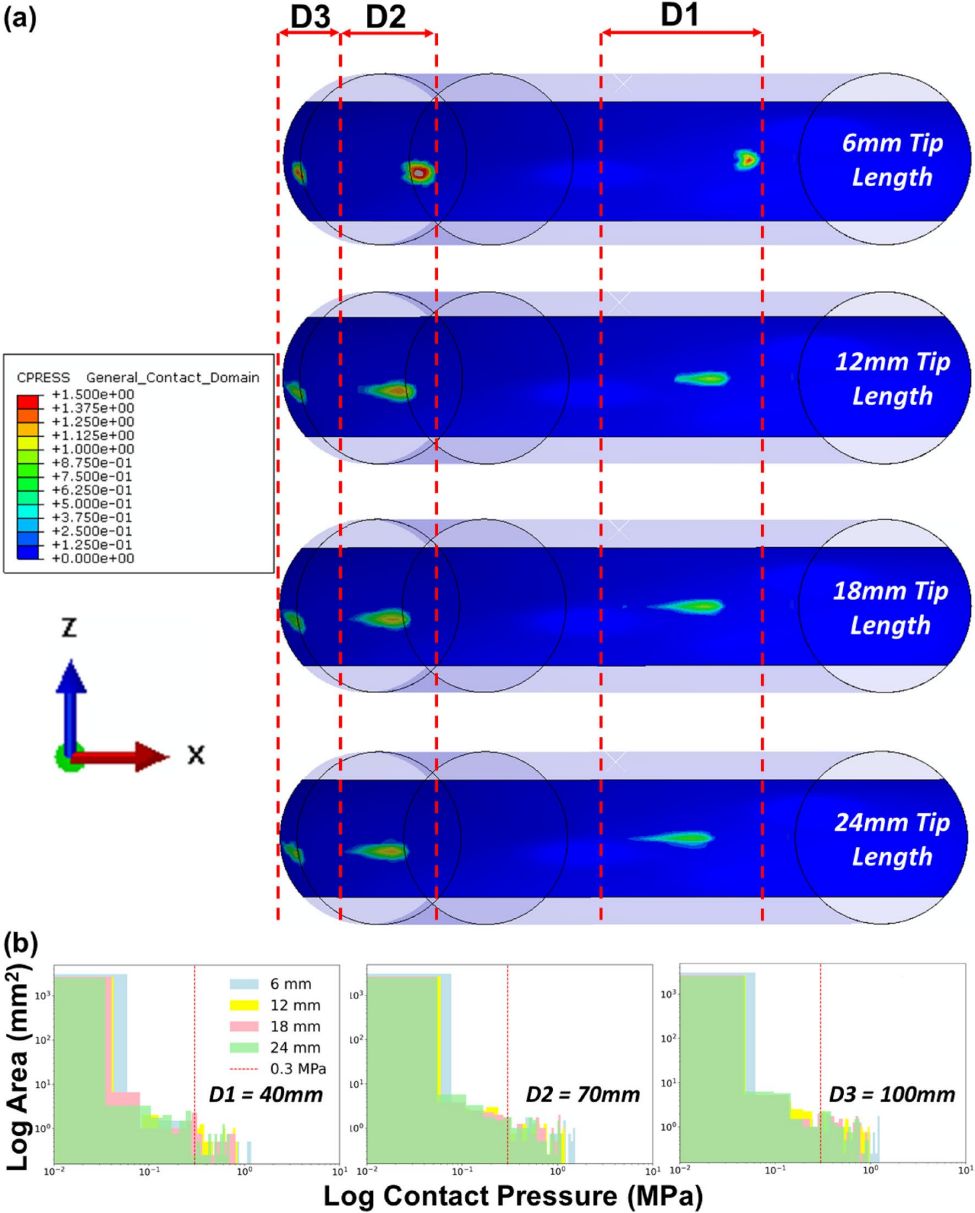
**a** Contour plots of contact pressure for each tip length (6, 12, 18 & 24 mm), overlaid at key displacement timepoints (D1–D3), see Fig. 5a*,* during catheter tracking within the idealized rigid aorta model; **b** Histograms of logarithmic tip contact pressure (CPRESS) per logarithmic element surface area of the rigid aorta wall at the displacement points depicted in Fig. 5a

**Fig. 8:**
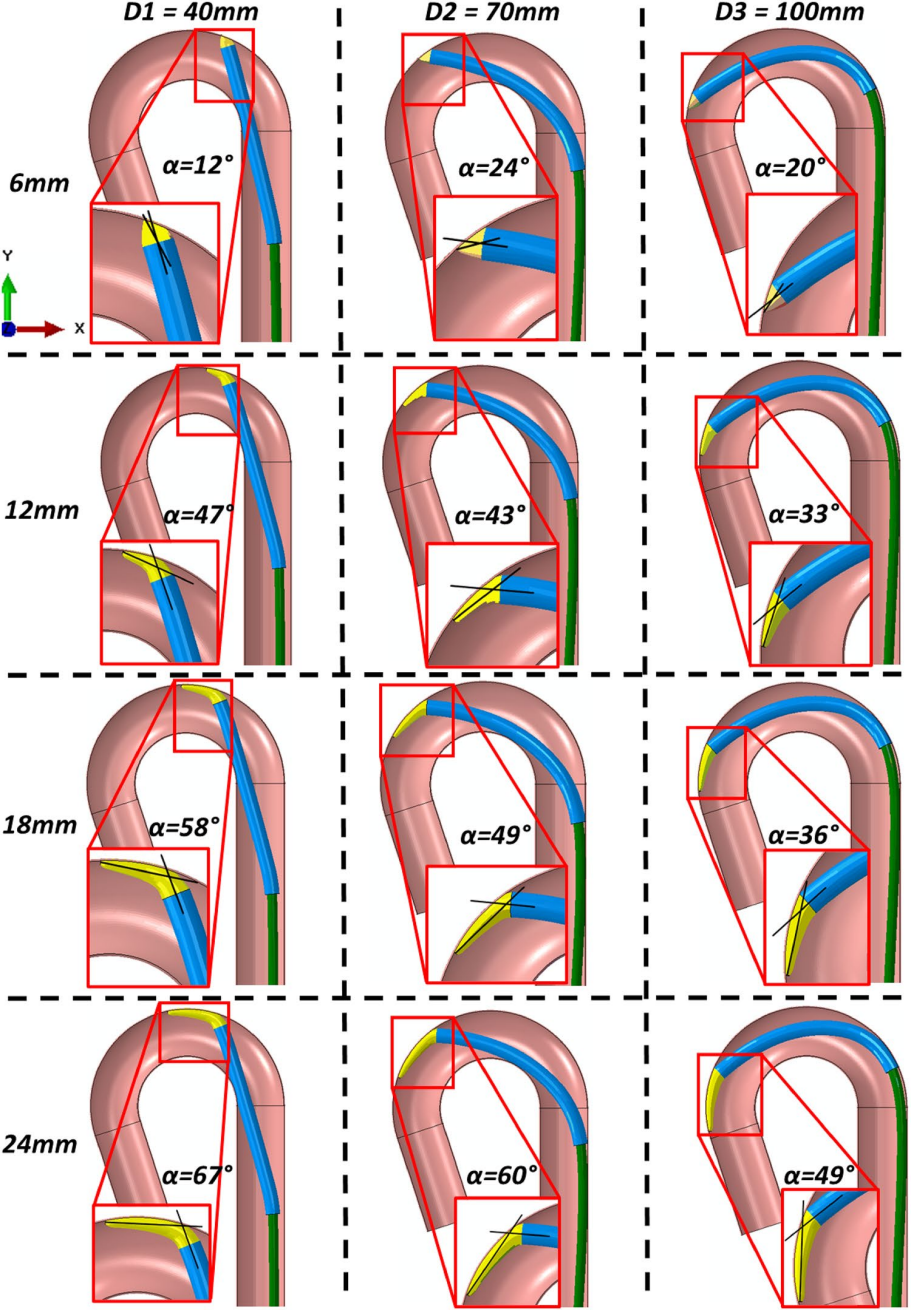
2D representation and tip angle of deflection measurements of varying idealized tip lengths (6–24 mm) during tracking at three key displacement points (D1–D3), see Fig. 5a

An investigation of the influence of catheter tip lengths on the contact pressures (Abaqus output variable plot of CPRESS) exerted on the aorta wall during catheter tracking was performed using the rigid aorta model. The predictions (Fig 7a, b) demonstrate that the shorter tip lengths increase the peak contact pressures on the aorta wall due to the smaller surface area and localisation of the contact. This was seen at all key displacement points. The contact pressure at displacement point D2 presents a 37 and 31% increase in peak contact pressure between the 6 mm tip length and the 18 and 24 mm tip lengths, respectively. This correlated with an increase of 33.3 and 42% in surface area under contact pressure > 0.3 MPa between the 6 mm tip length and the 18 and 24 mm tip lengths, respectively. Examining the contact pressures that exceed 1 MPa in these models indicates that 87.5% more surface area exceeds 1 MPa in the 6 mm tip length than in either the 18 mm or 24 mm tip lengths. The predictions shown in Fig. 8 reveal the variance in tip deflection angle for each tip length at each key displacement point. As expected, the predictions establish that shorter tip lengths correlated to smaller angles of deflection at each displacement point. The angle of tip deflection reduces as the catheter tracks around the arch which corresponds with an increase in contact pressure in all models except the 6 mm tip model.

## Discussion

In this study, experimental and FE methods were applied to examine and compare catheter tracking forces during TAVR delivery from in vitro benchtop testing and in-silico idealized aorta models. We report that a rigid trackability test model can provide a reasonable estimation of the peak reaction forces experienced during catheter tracking within a compliant aorta. In addition, we report that the stresses experienced in the plaque tissue during catheter tracking exceed the stress threshold for rupture of aortic arch wall tissue with plaque inclusions (300 kPa) for stiff plaque, soft plaque, and no plaque models. We highlight the potential risks associated with design change during catheter tracking and demonstrate that increasing the tip contact surface will reduce the peak contact pressures experienced in the tissue.

Previous studies have predominantly focused on in vitro testing to assess the tracking forces associated with catheter delivery systems for coronary stents [12, 39, 40]. There have been varying approaches for this in terms of in vitro vessel tortuosity, which can be defined by the amount of twist or arch curvature along the vessel course between both ends. Rieu et al. examined coronary delivery system trackability around two tortuosities comprised of simple 90 and 135° angles [12]. Szabadits et al. examined trackability through a more tortuous route with varying arch radii (30 and 15 mm) [39], while Finn et al. used patient-specific anatomy models for their trackability testing [40]. However, a gap in public knowledge remains in terms of the forces and stresses that TAVR catheter delivery systems impart on vessels during tracking. Studies by Sun et al. and Gunning et al. have implemented FE to investigate TAVR valve performance with regards to peak stresses on the leaflets [24, 25], while Mc Gee et al. and Bianchi et al. investigated implantation depth [41] and deployment geometry [42], respectively. Xiong et al. used computational methods to analyse the distribution of forces on the valve frame [43]. However, despite this extensive research into the valve performance, the stresses imparted on the vessel wall from the catheter delivery systems have not been investigated. The current study combines both in vitro and in-silico analyses to develop a modular idealized aorta model that can assess different biomechanical conditions and catheter design inputs. This methodology was expanded, with the addition of plaque inclusions to assess how the stresses imparted during catheter tracking on the aortic and plaque tissue could present a risk of rupture based on aortic and plaque tissue rupture stress thresholds.

There are some limitations to this study that require consideration. The rigid in-silico model analysed in this study under-predicts the reaction force peaks from in vitro catheter trackability testing, which might be attributed to factors that the in-silico model did not account for. One such factor might be the characterisation of the catheter shaft material properties. These were determined through 3-point bend-testing (and reproduced with FEA), for which it was assumed that the catheter shafts were homogenous. However, each shaft has a combination of braid layers and polymers, and this heterogeneous shaft combination may not be fully captured through 3-point bend-testing. This material assumption likely resulted in the disparity seen between in vitro and in-silico force-displacement curves. The in vitro benchtop model does not include the aortic head vessels in this study, which would have reduced the ease with which the catheter tracks around the aorta arch. The introduction of a guidewire through over-the-wire tracking would resolve any potential tracking issues created by the aortic head vessels. However, the guidewire was not included due to the computational cost and complexity of multiple contact surfaces. A preliminary in-silico analysis that included the guidewire was found to aid in catheter positioning while tracking around the arch but had no impact on peak reaction forces (data not shown).

Due to the idealized nature of these models, the morphology and geometrical variance of the aorta were not considered in these predictions. This was deemed necessary for efficient computational modelling of arterial mechanical behaviour under contact. The representation of the calcified plaques as homogenous could lead to an overestimation of the plaque’s stiffness in certain locations. Finally, including the plaques as fully embedded and idealized sections in the aorta may influence the predicted reaction forces. However, both the heterogeneity and morphology of the plaques were considered to be acceptable assumptions as the aim of this study was to efficiently investigate the role of plaque location and stiffness. In our study of catheter design, we did not include plaques. Nevertheless, we can infer that due to the variance in contact pressure (or compressive stress), the stresses imparted on the rigid wall do exceed the threshold for plaque rupture (300 kPa). However, the rigidity of the aorta wall in this prediction should be taken into account here. Future work will incorporate patient-specific anatomies to investigate more representative geometries.

Through implementing Kelly-Arnold et al.’s hypothesis for the explosive growth of voids within tissue leading to rupture, we find that there was no risk of diseased aortic arch tissue rupture based on this accepted aortic tissue stress threshold (4.1667 MPa) [23]. However, we report that both the aortic arch tissue and plaque tissue exceed the rupture stress threshold for aortic arch tissue with plaque inclusions (300 kPa) [20] for the stiff plaque, soft plaque, and no plaque models. It is important to note that the first analysis, which focused on Kelly-Arnold’s hypothesis regarding aortic tissue rupture, only analysed the aortic arch wall tissue in the stiff plaque, soft plaque and no plaque models (See Fig. 5a for differentiation of plaque and aortic arch wall tissue). As the plaques were excluded from the aortic arch tissue rupture analysis to examine the stresses in the vessel wall, and in particular, the vessel/plaque shoulder region, no aortic arch wall tissue was at risk of rupture per the higher threshold. The second analysis examined the same models, however, with the inclusion of the plaques with the aortic arch tissue to analyse both aortic and plaque tissue rupture risk. In this analysis we report a risk of plaque rupture between 0.5 and 1.5% of the element volume in the stiff plaque model at each catheter tracking point analysed (Fig. 6a). The soft plaque model exceeds the threshold at D2 (0.075%) and D3 (0.05%), while the no plaque model exceeds this threshold at displacement point D2 (0.065%) only. Interestingly, the presence of soft or stiff plaques in the aorta model did not significantly alter the peak reaction forces (Fig. 5b) when compared with the diseased homogenous aorta model (5 MPa). The predictions from this study reveal that catheter tracking produces a risk of plaque rupture in the embedded plaques, in particular, the stiff plaques. While the element volume at risk in the soft plaque and no plaque models is small, this still presents a risk to the patient and may result in micro-emboli formation leading to complications downstream. Implementing more representative geometries and morphologies through patient-specific anatomies could result in higher peak forces and stresses that could increase the risk of rupture in the soft or stiff plaques. Additionally, further research is needed to understand the mechanical properties of plaques as well as their stress rupture thresholds for reliable predictions of rupture risk.

This study also compared a rigid in-silico model with the biomechanically representative aorta model. The rigid model can still be a useful comparator to assess different biomechanical conditions despite the under-prediction (likely due to the material characterisation of the catheter shafts) presented in Fig. 3A. Interestingly, it was found that there was minimal variance in normalised peak reaction forces (0.37 v 0.35) between the models during catheter tracking (Fig. 4). This indicates that in terms of trackability testing, a stiff or rigid test model provides a reasonable estimation of the peak reaction forces experienced during tracking in softer and more compliant vessels.

It is interesting to understand whether catheter tip design can influence the reaction forces and rupture risk. Here we investigated the effects of catheter tip lengths (as shown in Figs. 7 and 8) and predicted that larger catheter tips provide a larger contact surface area, which results in lower peak contact pressures (Abaqus output variable plot of CPRESS) on the arterial wall during catheter tracking. Midway through catheter tracking, the 6 mm tip length had a peak contact pressure of 3.2 MPa, while the 24 mm tip length had a peak contact pressure of 1.15 MPa. The histograms presented in this investigation established that there was a 42% increase in the surface area of the aortic wall experiencing contact pressures greater than 0.3 MPa from the 6 mm to the 24 mm tip length. However, this relationship is flipped when contact pressures greater than 1 MPa are examined between the two tip lengths, with 87.5% more surface area in the 6 mm tip model exceeding 1 MPa when compared to the 24 mm tip model. These predictions confirm that increasing the surface area experiencing contact with the larger tip lengths will reduce the peak contact pressures experienced in the tissue. In addition, the predictions in Fig. 8 reveal that the increases in contact pressures seen in the shorter tip lengths also correspond to smaller angles of deflection, illustrating the correlation between tip angle deflection and contact pressure. These findings have implications for the medical device industry and physicians as they underline the potential risks associated with shorter catheter tip lengths during catheter tracking.

In conclusion, the effect of TAVR catheter delivery systems during catheter tracking on the stresses and contact pressures imparted on idealized aorta walls was investigated for the first time in this study. Computational modelling was used to assess the risk of rupture of aortic wall tissue with and without plaque inclusions. This study has found that a small proportion of plaque and aortic arch tissue is at risk of rupture (1.5%) during catheter tracking when compared with a rupture stress threshold of 0.3 MPa [20]. However, when investigating only the aortic wall tissue, no tissue is found to be at risk when examined with the higher threshold for rupture of 4.2 MPa [21–23]. This emphasises the challenges faced in predicting the risk of vessel injury or plaque rupture occurring through FE methods based on the current scientific knowledge. Computational modelling can be a useful tool in the predictive analysis of TAVR delivery once the materials and their mechanical behaviours are fully understood. The predictions in this study highlight a small risk of plaque rupture, due to catheter tracking, with future work planned to incorporate realistic plaque morphologies to add to this knowledge.
